# Undiagnosed Thyrotoxicosis Storm in a Patient Presenting With a Perioperative Sinus Tachycardia and Hypertensive Crisis After Induction of Anesthesia for Emergent Bipolar Hip Hemiarthroplasty After Traumatic Femur Neck Fracture: A Case Report

**DOI:** 10.7759/cureus.31145

**Published:** 2022-11-06

**Authors:** Eyad F Alsayed

**Affiliations:** 1 Department of Anesthesia & Critical Care, King Abdulaziz University Faculty of Medicine, Jeddah, SAU; 2 Anesthesia Services Division, King Abdulaziz University Hospital, Jeddah, SAU

**Keywords:** drug induced sympathetic activation, perioperative hypertension, perioperative tachycardia, thyrotoxicosis, thyroid storm

## Abstract

The clinical picture of sympathetic overdrive under anesthesia can be caused by many syndromes, which should be considered and excluded. Thus, the anesthetist needs a high level of suspicion and vigilance to recognize the diagnosis early and act accordingly. Emphasizing the pitfalls encountered in this exceptional case scenario, we present this rare case of a malnourished patient on antipsychotic medications facing an urgent operation concomitant with a thyroid storm, a situation that can rapidly progress to a decompensating multiorgan failure.

## Introduction

The clinical presentation of thyrotoxicosis ranges from abnormally elevated serum thyroid hormone levels to a severe catabolic crisis with multiorgan failure. In stressful situations, the patient's ability to maintain adequate metabolic, thermoregulatory, and cardiovascular compensatory mechanisms might be compromised, leading to a thyroid crisis or storm [1]. This makes it crucial to be diagnosed early based on high clinical suspicion even before confirmatory biochemical tests, as it can easily lead to fatal progress.

## Case presentation

A 56-year-old female patient presented to the ER with a history of tripping and falling on her left hip in her house. With the help of family members, she was seated in a wheelchair because of restrictive, painful mobility. On examination, she showed an externally rotated and shortened lower limb. This clinical picture raised the suspension of the femur neck fracture. The diagnosis was confirmed with a plain radiograph, as the femur shaft was displaced.

The patient had a long history of type II diabetes mellitus, complicated by a neuropathic arthropathy (Charcot joint) of the left foot. Therefore, it mandates multiple reconstructive and stabilizing surgical interventions. After performing a gastric sleeve operation 10 years ago, the diabetes was controlled, and there was no need to continue on anti-diabetic medications. In the last three years, the patient started to experience psychiatric problems in the form of depressive episodes and delusions. Lately, her psychiatrist prescribed quetiapine and olanzapine in addition to low-dose propranolol for her familial benign tremors. This course of the psychiatric disease was accompanied by a loss of appetite, which led to a severe reduction in weight, malnutrition state, and cachexia, weighing only 46 kg on presentation with a height of 176 cm (BMI = 15.2 kg/m2).

The orthopedic team indicated a hemiarthroplasty hip replacement as optimal surgical management in her case. Her blood investigations revealed a hemoglobin of 7.8 g/dL on admission. She was transferred two erythrocytes concentrate prior to surgery to optimize her cardiocirculatory situation.

Aside from her cachectic and anxious appearance, she was vitally stable on presentation to the operating room with light tachycardia with a heart rate of 95 bpm, a slightly elevated blood pressure of 130/88 mmHg, and a temperature of 36.7°C. She was premedicated with midazolam. The induction of anesthesia was performed with propofol, fentanyl, and rocuronium. Sevoflurane was used for the maintenance of the anesthesia. After induction of anesthesia, the patient started to be tachycardic to 147 bpm and developed severe hypertension of 240/130 mmHg.

With the absence of muscle rigidity, stable partial pressure of carbon dioxide (PaCO2), and uneventful multiple previous exposures to anesthesia for variant surgeries, malignant hyperthermia was at the bottom list of our differential diagnosis. Neuroleptic malignant syndrome, serotonin release syndrome, and thyroid storm precipitated by surgery were not to be excluded in this case. Although this is not the typical presentation of pheochromocytoma, it can manifest as a first episode under anesthesia in a previously normotensive patient. Supportive measures and symptomatic management were performed with beta-blockers and antihypertensive medication.

The highly experienced surgeon performed the case in 20 minutes. The patient was extubated immediately in the theatre with no recorded adverse events and transferred to prolonged observation in the recovery room.

In the recovery room, the thyroid panel was sent to the laboratory, and the results showed elevated serum thyroid hormone levels (free T3 level: 6.17 pmol/L, serum fT4: 43.31 pmol/L) and undetectable thyroid stimulating hormone (TSH) (<0.001 μIU/ml) consistent with thyroid storm.

## Discussion

Even though the mechanism is not clearly understood, a thyroid storm exhibits most of its clinical picture because of sympathetic overactivity. Many mechanisms were proposed, including and not limited to, an increased number of adrenergic receptors on target organs and a poorly understood post-receptor event that increases the catecholamines sensitivity, which ends with the induction of key enzymes regulating metabolism [2].

Many patients would present to the operating room with a history of either recently developed or already diagnosed and therapied thyrotoxicosis, which eases the anticipation of thyroid storm. This was not the case with our patient. Retrospectively, it becomes evident that the disease might have had an insidious onset in the last few years and played role in weight loss, osteoporosis, and psychiatric illness. It is worth mentioning that the association between hyperthyroidism and osteoporosis is well-established [3,4].

However, the first syndromes that were suspected as the patient started to be tachycardic and hypertensive were serotonin syndrome and malignant neuroleptic syndrome, as the patient was on antipsychotic medications in the last three years. With the absence of muscle rigidity, stable PaCO2, and uneventful multiple previous exposures to anesthesia for variant surgeries, malignant hyperthermia was considered less likely to be the causative syndrome. Pheochromocytoma is another important diagnosis that can be triggered by mechanical or psychological stress or after induction of anesthesia, even without stressful manipulation, as reported by Sonntagbauer et al. [5].

Although fever is a defining symptom of neuroleptic malignant syndrome, it may not be evident in cases inherent with atypical antipsychotics [6]. The fulminant form typically includes a high fever ≥ 40°C and muscle rigidity. Followed by a wide range of mental status changes (from drowsiness to severe delirium and coma with or without seizures), tachycardia and tachypnea, rhabdomyolysis and acute renal failure, elevated lactate and liver functions tests, and leukocytosis are all features of the syndrome [7]. Immediate discontinuation of the causative agent and supportive measures are the mainstay of the management. The muscle relaxant dantrolene might help, and re-initiating an abruptly discontinued dopaminergic agent is recommended [8].

In serotonin syndrome, the mechanism might be complex and multifarious. The surging levels of serotonin in the brain's neuronal junctions cause central catecholamine release [9]. The end result is activating the sympathetic nervous system through different mechanisms, including anterior hypothalamus stimulation and secretion of thyroid hormones and cortisol [10]. This leads to CNS hyperreflexia, with symptoms ranging in mild cases from tremors and diarrhea and increasing in severity into delirium, muscular rigidity, and hyperthermia in severe cases [10].

Pheochromocytoma would be diagnosed in the outpatient setting as the patients experience bursts of hypertension episodes associated with sweating, palpitations, and headache secondary to abrupt catecholamine release [11]. Nevertheless, and mainly through the high interindividual differences in catecholamines sensitivity, there is a poor correlation between the levels of circulating catecholamines and the symptomatic complaints. Around 13% of the patients might have normal blood pressure, and 48% might only experience paroxysmal episodes of hypertension [12].

In some case reports, the active paragangliomas were described in the literature as an incidental finding. It was mainly in cases where an abnormal mass was planned to be excised. In this case, manipulating the mass can cause massive hemodynamic instability and cardiovascular fluctuations [13,14]. However, a catecholamine crisis due to pheochromocytoma can also issue under anesthesia in a previously normotensive patient [5]. Exclusion of the diagnosis with the appropriate tests should be sought.

As the diagnosis was confirmed as thyroid storm with laboratory tests in the recovery room, the patient was treated accordingly. The treatment of thyroid storm can be categorized into targeting thyroid hormone synthesis and secretion, suppressing the peripheral conversion and activity of triiodothyronine at the tissue level, reversal of the systematic decompensation, and treatment of the precipitating factor [15]. The definitive treatment should follow the acute phase after stabilization. If the patient was previously known to be hyperthyroid, it was strongly recommended in the American Thyroid Association guidelines to yield the patient euthyroid before the procedure with antithyroid medications with a combination of a β-adrenergic blocker or not. If thyroidectomy was planned for the treatment of Graves’ disease, thyroid iodine uptake blockade with potassium iodide supplementation in the immediate perioperative period was also advised [15].

Medication selection also plays an important role. Selecting propranolol over the other β-blocker might be beneficial as it hinders the monodeiodinase-type-I enzyme, which transforms T4 into the more biologically functional T3 form of the hormone [16]. Glucocorticoids also decrease the transformation of T4 to T3 within a couple of hours and belong to the therapy regime of thyroid storm [16].

It is worth mentioning that the treatment of moderate to severe hyperthyroidism prior to urgent surgeries, although must be attempted to decrease the chance of developing thyroid storm, it does not always prevent it. In a systematic review by de Mul et al., the risk of the perioperative storm in hyperthyroid patients was still present even in a cohort treated with a triple combination of medication before the operation [17]. Thus, the anesthetist must stay vigilant and prepared to deal with the situation at any given time perioperatively.

## Conclusions

We have presented a complicated case of a thyroid storm. In the first few seconds after the induction of anesthesia, as the patient started to be tachycardic, dehydration was suspected considering the malnutrition state of the patient. As hypertension became evident, we had to broaden our differential to include all causes of sympathetic overactivity. We hope we could presented a case that adds educational value for any member who might face such a scenario in the perioperative period. Preparedness and careful going through the deferential diagnosis are critical factors in managing such a crisis.
